# β-Asarone Ameliorates β-Amyloid–Induced Neurotoxicity in PC12 Cells by Activating P13K/Akt/Nrf2 Signaling Pathway

**DOI:** 10.3389/fphar.2021.659955

**Published:** 2021-05-10

**Authors:** Miaomiao Meng, Lijuan Zhang, Di AI, Hongyun Wu, Wei Peng

**Affiliations:** ^1^ The Second Clinical College, Guangzhou University of Chinese Medicine, Guangzhou, China; ^2^ Department of Clinical Education Management, The Affiliated Hospital of Shandong University of Traditional Chinese Medicine, Jinan, China; ^3^ College of Traditional Chinese Medicine, Shandong University of Traditional Chinese Medicine, Jinan, China; ^4^ Department of Neurology, The Affiliated Hospital of Shandong University of Traditional Chinese Medicine, Jinan, China

**Keywords:** β-asarone, Alzheimer’s disease, oxidative stress, P13K/Akt/Nrf2 signaling pathway, Aβ1-42

## Abstract

Accumulation of β-amyloid (Aβ) causes oxidative stress, which is the major pathological mechanism in Alzheimer’s disease (AD). β-asarone could reduce Aβ-induced oxidative stress and neuronal damage, but the molecular mechanism remains elusive. In this study, we used an Aβ-stimulated PC12 cell model to explore the neuroprotective effects and potential mechanisms of β-asarone. The results showed that β-asarone could improve cell viability and weaken cell damage and apoptosis. β-asarone could also decrease the level of ROS and MDA; increase the level of SOD, CAT, and GSH-PX; and ameliorate the mitochondrial membrane potential. Furthermore, β-asarone could promote the expression of Nrf2 and HO-1 by upregulating the level of PI3K/Akt phosphorylation. In conclusion, β-asarone could exert neuroprotective effects by modulating the P13K/Akt/Nrf2 signaling pathway. β-asarone might be a promising therapy for AD.

## Introduction

Alzheimer’s disease (AD) is the most common neurodegenerative disease and the primary cause of dementia in elderly people. The neuropathology of AD includes accumulation of β-amyloid (Aβ), neurofibrillary tangles, and loss of neurons (Scarpini et al., 2003). Numerous pieces of evidence suggested that mitochondria dysfunction and oxidative stress were involved in AD pathogenesis and progression (Wang et al., 2014; Tobore, 2019). Mitochondria dysfunction is one of the earliest detected pathogenic changes observed in AD (Rhein et al., 2009; Yao et al., 2009). Previous studies showed that the deposition of Aβ caused the production of reactive oxygen species (ROS) and mitochondria dysfunction, which can induce oxidative stress and neuronal apoptosis (Habib et al., 2010; Huang et al., 2012; Butterfield et al., 2013; Bhat et al., 2015). Therefore, it might be helpful to inhibit Aβ-mediated oxidative stress for the prevention and treatment of AD.

Nuclear factor erythroid 2–related factor 2 (Nrf2) is a transcription factor that plays an important role in regulating oxidative stress. Accumulating studies showed that the activation of Nrf2 exerted neuroprotective activity via reducing oxidative stress injury in AD (Khodagholi et al., 2010; Liang et al., 2019). Moreover, Nrf2 also defended mitochondria from oxidative damage through decreasing overproduction of ROS (Brandes and Gray, 2020). Phosphoinositide 3-kinase (PI3K)/protein kinase B (Akt) signaling was considered a master upstream pathway of Nrf2, which could regulate oxidative stress by modulating the Nrf2/HO-1 pathway (Xi et al., 2012; Zhang et al., 2017; Zhao et al., 2020). Thus, the P13K/Akt/Nrf2 pathway was regarded as a potential treatment strategy against AD.

*Acorus tatarinowii* Schott could ameliorate cognitive deficit via controlling oxidative stress damage and was widely used in the treatment of AD (Esfandiari et al., 2018; Zhu et al., 2019). β-asarone is the main component of *Acorus tatarinowii* Schott volatile oil, which possesses diverse pharmacological properties such as anti-inflammatory, anti-oxidant, anti-apoptosis properties, and so on. *In vivo* and *in vitro* studies of AD showed that β-asarone could suppress Aβ by regulating autophagy (Wang et al., 2019; Deng et al., 2020). A recent study demonstrated that β-asarone could reduce Aβ-induced oxidative stress and neuronal damage (Saki et al., 2020). However, the protective mechanism of β-asarone has not been well-illuminated.

In this study, we focused on the effect of β-asarone on the cell apoptosis, ROS, and mitochondria dysfunction in PC12 cells. Furthermore, we explored the effect of β-asarone on Aβ-induced oxidative damage and attempted to elucidate the underlying mechanism.

## Materials and Methods

### Chemicals

β-asarone, Aβ_1–42_, and 3-[4, 5-dimethylthiazol-2-yl]-2, 5-diphenyl tetrazolium bromide (MTT) were obtained from Sigma-Aldrich Inc. (St. Louis, MO, United States). Dulbecco’s modified Eagle’s medium (DMEM), fetal bovine serum (FBS), 100 U/ml penicillin, and 100 μg/ml streptomycin were obtained from Hyclone (Thermo Fisher Scientific, WLM, Mass, United States). Phosphate-buffered saline (PBS) was obtained from Nanjing SunShine Biotechnology Co., Ltd. (Nanjing, China). A total superoxide dismutase (SOD) assay kit, lipid peroxidation malondialdehyde (MDA) assay kit, catalase (CAT) assay kit, total glutathione peroxidase (GSH-PX) assay kit, lactate dehydrogenase (LDH) cytotoxicity assay kit, and 2′, 7′-dichlorofluorescin diacetate (DCFH-DA) probe were purchased from Beyotime Biotechnology (Shanghai, China). An apoptosis detection kit was purchased from Thermo Fisher Scientific (WLM, Mass, United States). MitoSOX Red Mitochondrial Superoxide Indicator was purchased from Yeasen Biotech Co., Ltd. (Shanghai, China). A mitochondrial membrane potential assay kit with JC-1 was obtained from Solarbio (Beijing, China). Antibodies against HO-1 were obtained from Gene Tex Inc. (SA, Texas, United States). Antibodies against Nrf2, Bax, Bcl-2, cleaved caspase-3, P13K, P-P13K, P-Akt, Akt, β-actin, and Lamin A were obtained from Abcam (Cambridge, United Kingdom).

### Cell Culture and Drug Preparation

Rat pheochromocytoma (PC12) cells were obtained from the Chinese Academy of Sciences, cultured in Dulbecco’s modified Eagle’s medium (DMEM), and supplemented with 10% FBS, 100 U/ml penicillin, and 100 μg/ml streptomycin in a humidified atmosphere at 37°C with 5% CO_2_. Aβ_1–42_ was dissolved in PBS and incubated at 37°C for 7 days to induce aggregation (Tong et al., 2018; Zhu et al., 2019). Then, the aggregated Aβ_1–42_ was diluted to the desired concentration. β-asarone was dissolved to a concentration of 10 mM in dimethyl sulfoxide (DMSO). Working solutions were diluted from the stock solutions at a suitable concentration for further use.

### MTT Assay

Cell viability was tested by using MTT assay. The PC12 cells were plated in 96-well plates, with 1 × 10^4^ cells per well, and cultured for 24 h. After the cells were treated with β-asarone or Aβ_1–42_ or coprocessing, MTT (20 μl, 5 mg/ml) was added in each well and incubated for 4 h at 37°C. Then, the medium was removed and replaced with 150 μl/well of DMSO. The formazan crystals were dissolved, and the optical density was measured at 570 nm by using a microplate reader (Thermo Fisher Scientific, WLM, Mass, United States).

### LDH Assay

Cell damage was determined by using an LDH cytotoxicity assay kit. The PC12 cells were seeded in 96-well plates (1 × 10^4^ cells/well). The LDH level was measured according to the manufacturer’s protocol. The absorbance was determined using a microplate reader at 450 nm.

### Flow Cytometry Analysis of Apoptosis

Cell apoptosis was detected by using an apoptosis detection kit according to the manufacturer-recommended protocol. In brief, PC12 cells were cultured in six-well plates (1 × 10^6^ cells/well) for 24 h. After exposure to 10 μM Aβ_1–42_ for 12 h and treatment with β-asarone (0, 10, 30, and 60 μM) for another 12 h, the cells were washed and suspended in 100 μl binding buffer. Then, Annexin V-FITC and propidium iodide (PI) were added and incubated for 15 min at room temperature. The cells were analyzed by flow cytometry (BD Biosciences, San Jose, CA, United States).

### Measurement of Mitochondrial Membrane Potential

The mitochondrial membrane potential was detected by using the JC-1 assay. PC12 cells were incubated in 24-well plates, with 1 × 10^4^ cells per well. After pretreatment with Aβ_1–42_ for 12 h, the cells were treated with various concentrations of β-asarone for another 12 h. Then, the cells were stained with the JC-1 dye for 20 min at 37°C. Positive JC-1 staining was identified by fluorescence microscopy.

### Assay of Oxidative Biochemical Parameters

The PC12 cells were seeded into six-well plates, with 4 × 10^5^ cells per well. After pretreatment with Aβ_1–42_ for 12 h and incubation with various concentrations of β-asarone for another 12 h, the cells were collected and lysed. Then, the mixture was centrifuged at 14,000 g for 10 min, and the supernatant was used for the assay. Then, protein concentration was determined by using a BCA assay kit. The SOD, MDA, CAT, and GSH-PX activities were measured according to the kit instructions.

### Measurement of ROS Levels

The accumulation of ROS was monitored by using a fluorescent probe, 2’, 7’-dichlorodihydrofluorescin diacetate (DCFH-DA). In brief, the PC12 cells were cultured in six-well plates (4 × 10^5^ cells/well). After pretreatment with Aβ_1–42_ for 12 h, the cells were treated with various concentrations of β-asarone for another 12 h. Then, the cells were washed with PBS and stained with 1 μM DCFH-DA for 20 min. Then, the cells were washed three times with PBS, and the fluorescence intensity was detected with a fluorescence microscope (Carl Zeiss Co., Ltd., Shanghai, China).

PC12 cells were incubated with 5 μM MitoSOX Red and 10 μg/ml of the nuclear staining dye Hoechst for 20 min at 37°C. The fluorescent signals were recorded by using a confocal laser scanning microscope (Carl Zeiss Co., Ltd., Shanghai, China).

### Western Blot Analysis

The PC12 cells were lysed with RIPA buffer after treatment. Then, protein concentration was quantified by using a BCA kit. Proteins were separated by 10% SDS-PAGE electrophoresis and transferred to polyvinylidene difluoride (PVDF) membranes. The membranes were blocked with 5% nonfat milk for 1 h at room temperature and incubated with primary antibodies against HO-1, P13K, P-P13K, P-Akt, Akt, Bax, Bcl-2, cleaved caspase-3, Nrf 2, and β-actin (1:1000) at 4°C overnight. Subsequently, the membranes were incubated with secondary antibodies (1:5000) at 37°C for 2 h. The bands were visualized with enhanced chemiluminescence reagent (ECL) under a Tanon-5200 Chemiluminescent Imaging System (Tanon Science & Technology Co., Ltd., Shanghai, China).

### Statistical Analysis

The data were analyzed with SPSS 22.0 statistic program, and the results were expressed as means ± SD by one-way ANOVA and Turkey’s post hoc test. The value of *p* < 0.05 was considered statistically significant.

## Results

### Effect of β-asarone on Aβ_1–42_-Induced Cytotoxicity in PC12 Cells

**FIGURE 1:**
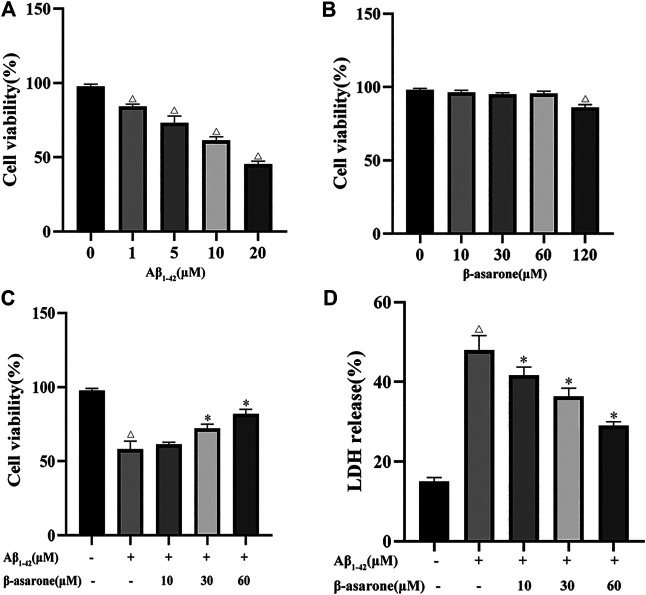
Effect of β-asarone on Aβ_1–42_-induced cytotoxicity. PC12 cells were treated with different doses of Aβ_1–42_ (0, 1, 5, 10, and 20 μM) for 12 h **(A)** or β-asarone (0, 10, 30, 60, and 120 μM) for 12 h **(B),** and cell viability was detected by using the MTT assay. PC12 cells were treated with 10 μM Aβ_1–42_ for 12 h, followed by β-asarone treatment for another 12 h, and subjected to MTT assay **(C)** and LDH release tests **(D)**. ^△^ *p* < 0.05 compared with the control group; ^*^ *p* < 0.05 compared with the Aβ_1–42_-alone treatment group. Each column represents mean ± SD, and each experiment was repeated three times.

To determine the proper concentration ranges for the study, we first investigated the effect of β-asarone and Aβ_1–42_ alone on PC12 cell proliferation. As shown in Figures 1A,B, Aβ_1–42_ significantly inhibited PC12 cell viability at a concentration range of 1–20 μM (*p* < 0.01), and the 50% inhibitory concentration was about 10 μM. β-asarone (0–60 μM) showed no significant effect on the proliferation of PC12 cells. When the concentration was increased to 120 μM, the cell viability decreased (*p* < 0.05). We selected 10 μM Aβ_1–42_ and 0–60 μM β-asarone for subsequent experiments. Further study revealed that the cell viability was significantly increased (62.83, 73.75, and 83.98% of the control value, respectively) by β-asarone treatment. The better protective effect was shown at a concentration of 60 μM (Figure 1C).

The release of LDH can reflect cell damage and cytotoxicity. Furthermore, the LDH level of Aβ_1–42_-induced PC12 cells was measured. The results showed that the LDH release increased after Aβ_1–42_ treatment (*p* < 0.05). When cells were treated with β-asarone, the LDH release decreased (*p* < 0.05). The results indicated that β-asarone could reduce Aβ_1–42_-induced cell cytotoxicity in a concentration-dependent manner (Figure 1D).

### Effect of β-Asarone on Aβ_1–42_-Induced Apoptosis in PC12 Cells

**FIGURE 2:**
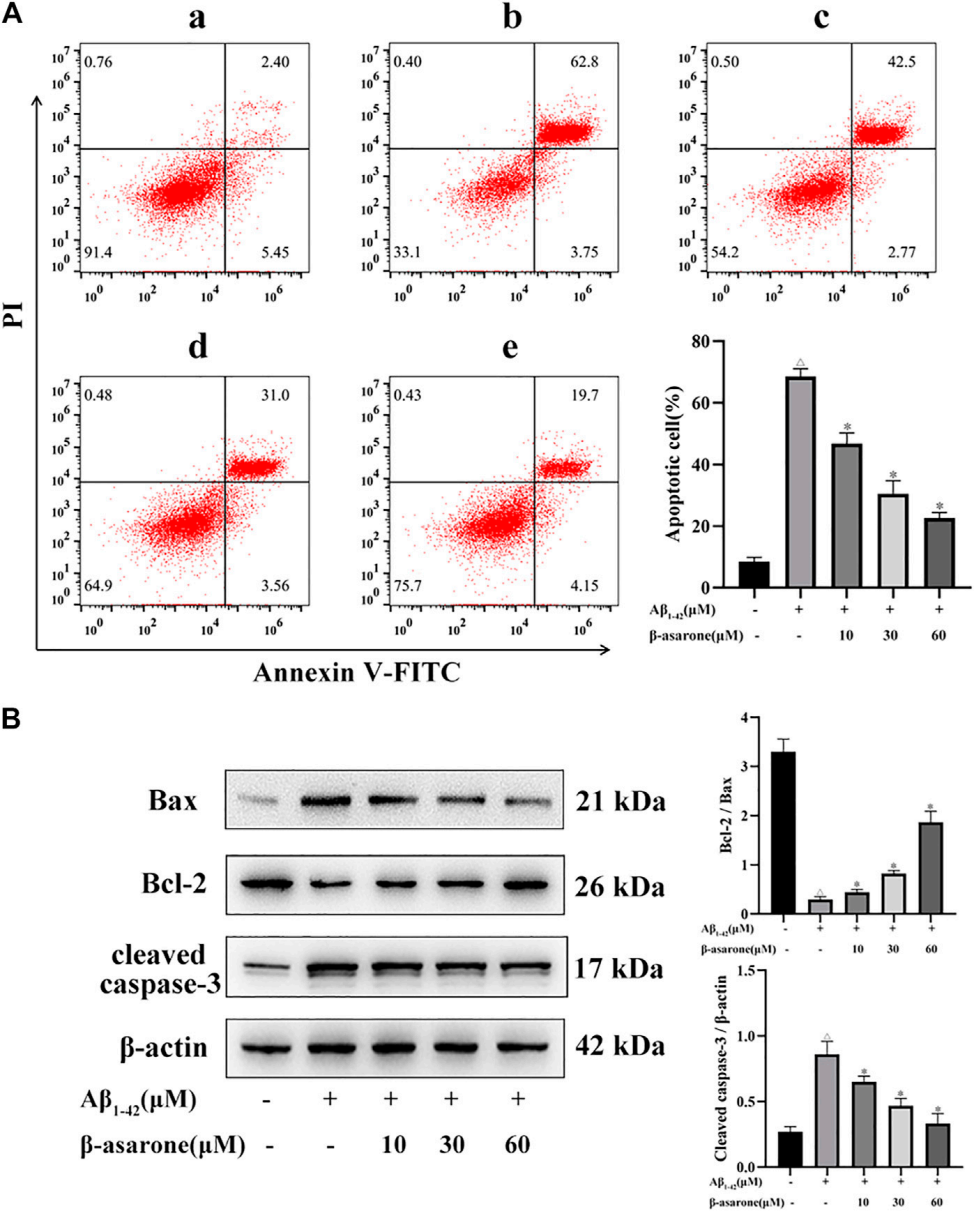
Effect of β-asarone on apoptosis in PC12 cells. **(A)** Apoptosis was measured by flow cytometry, and the percentage of early and late apoptotic cells was quantified. The left lower quadrant represents the normal cells, the right lower quadrant represents the early apoptosis cells, the right upper quadrant represents the late apoptosis cells, and the left upper quadrant represents the death cells. a, Control group; b, Aβ_1–42_-alone treatment group; c, Aβ_1–42_+β-asarone (10 μM) group; d, Aβ_1–42_+β-asarone (30 μM) group; e, Aβ_1–42_+β-asarone (60 μM) group. **(B)** Expression levels and quantification of Bcl-2/Bax and cleaved caspase-3 were examined by Western blot. ^△^ *p* < 0.05 compared with the control group; ^*^ *p* < 0.05 compared with the Aβ_1–42_-alone treatment group. The data were expressed as mean ± SD of three independent experiments, each in triplicate.

To explore the effect of β-asarone on Aβ_1–42_-induced apoptosis, flow cytometry analysis and Western blot analysis were used. As shown in Figure 2A, Aβ_1–42_ could increase cell apoptosis (*p* < 0.05). However, compared with treatment with Aβ alone, treatment with β-asarone could decrease apoptosis in a dose-dependent manner (*p* < 0.05). Furthermore, the expressions of Bcl-2, Bax, and cleaved caspase-3 were tested. The results showed that treatment with Aβ_1–42_ alone could decrease the ratio of Bcl-2/Bax slightly and increase the level of cleaved caspase-3. However, β-asarone could reverse these markers markedly (Figure 2B; *p* < 0.05).

### Effect of β-Asarone on Aβ_1–42_-Induced Mitochondrial Membrane Potential in PC12 Cells

**FIGURE 3:**
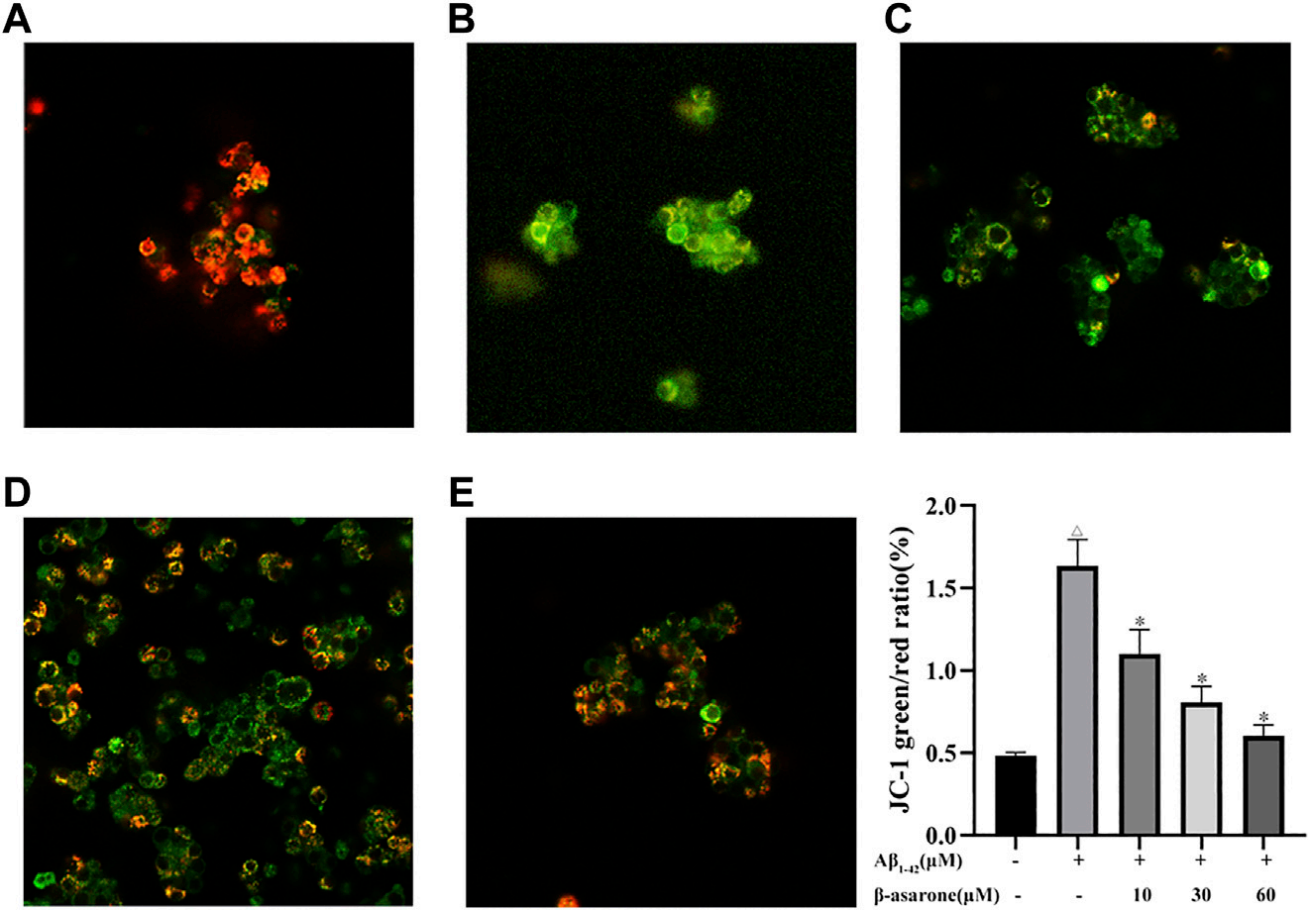
Effect of β-asarone on Aβ_1–42_–induced mitochondrial membrane potential. PC12 cells were stained with JC-1 and observed under a fluorescence microscope (× 400). **a**, control group; **b**, Aβ_1–42_ alone treatment group; **c**, Aβ_1–42_+β-asarone (10 μM) group; **d**, Aβ_1–42_+β-asarone (30 μM) group; **e**, Aβ_1–42_+β-asarone (60 μM) group; ^△^ *p* < 0.05 compared with the control group; ^*^ *p* < 0.05 compared with the Aβ_1–42_ alone treatment group. The data were expressed as mean ± SD from three independent experiments.

To assess the effect of β-asarone on Aβ_1–42_-induced mitochondrial membrane potential, JC-1 assay was used. When cells were treated with Aβ alone, the green fluorescence increased. The results implied that Aβ induced the loss of the mitochondrial membrane potential. After β-asarone treatment, the fluorescence shifted from green to red in a concentration-dependent manner. Hence, the reduction of the mitochondrial membrane potential could be reversed by β-asarone treatment (Figure 3; *p* < 0.05).

### Effect of β-Asarone on Aβ_1–42_-Induced Oxidative Stress in PC12 Cells

**FIGURE 4:**
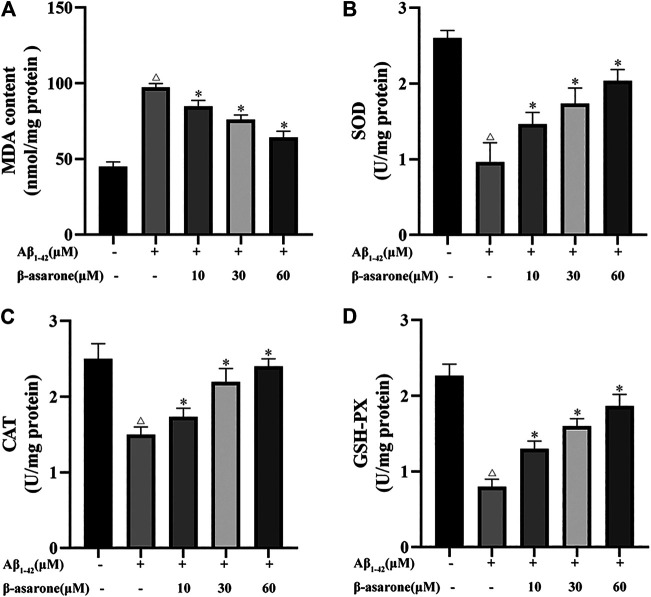
Effect of β-asarone on Aβ_1–42_-induced oxidative stress. **(A)** Effect of β-asarone on Aβ_1−42_-induced MDA levels. **(B)** Effect of β-asarone on Aβ_1−42_-induced SOD levels. **(C)** Effect of β-asarone on Aβ_1−42_-induced CAT levels. **(D)** Effect of β-asarone on Aβ_1−42_-induced GSH-PX levels. ^△^ *p* < 0.05 compared with the control group; ^*^ *p* < 0.05 compared with the Aβ_1–42_-alone treatment group. Data from three independent experiments were expressed as mean ± SD.

To observe the effect of β-asarone on Aβ_1–42_-induced oxidative stress, the levels of MDA, SOD, CAT, and GSH-PX were measured. After exposure to 10 µM Aβ_1–42_, the MDA level was increased (Figure 4A; *p* < 0.05) and the levels of SOD, CAT, and GSH-PX were suppressed compared with the control group (Figures 4B–D; *p* < 0.05). Nevertheless, the levels of MDA, SOD, CAT, and GSH-PX were noticeably reversed by different concentrations of β-asarone treatment (*p* < 0.05).

### Effect of β-Asarone on Aβ_1–42_-Induced ROS Production in PC12 Cells

**FIGURE 5:**
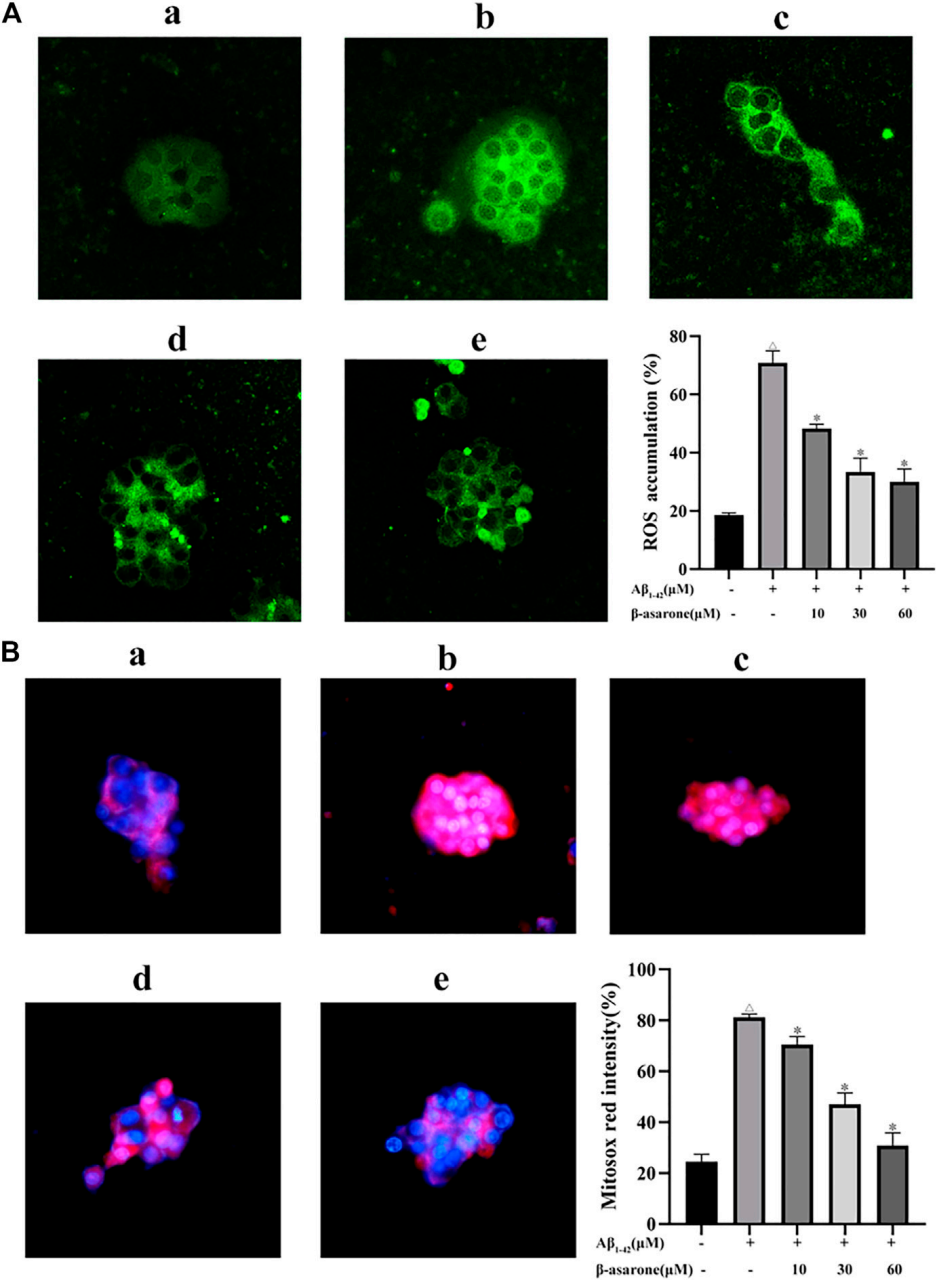
Effect of β-asarone on Aβ_1–42_-induced ROS production. After PC12 cells were treated, the intracellular ROS level was observed using a fluorescence microscope (× 400) and detected using a fluorescence microplate reader **(A)**. **(B)** The mitochondrial ROS generation was observed using MitoSOX specific red dye (red color). a, Control group; b, Aβ_1–42_-alone treatment group; c, Aβ_1–42_+β-asarone (10 μM) group; d, Aβ_1–42_+β-asarone (30 μM) group; e, Aβ_1–42_+β-asarone (60 μM) group. ^△^ *p* < 0.05 compared with the control group; ^*^ *p* < 0.05 compared with the Aβ_1–42_-alone treatment group. Data from three independent experiments were expressed as mean ± SD.

To examine the effect of β-asarone on Aβ_1–42_-induced ROS production, DCFH-DA fluorescein-labeled dye was used. After Aβ_1–42_ treatment, the green fluorescence was enhanced. The results confirmed that the intracellular ROS level was increased by Aβ treatment (Figure 5A; *p* < 0.05). However, β-asarone could inhibit Aβ_1–42_-induced ROS accumulation. Next, mitochondrial ROS generation was measured using MitoSOX specific red dye. As shown in Figure 5B, β-asarone weakened the red fluorescence compared with the Aβ_1–42_-alone treatment group (*p* < 0.05). Thus, β-asarone could alleviate Aβ_1–42_-induced mitochondrial superoxide generation.

### Effect of β-Asarone on the Protein Expression of P13K/Akt/Nrf2/HO-1 in PC12 Cells

**FIGURE 6:**
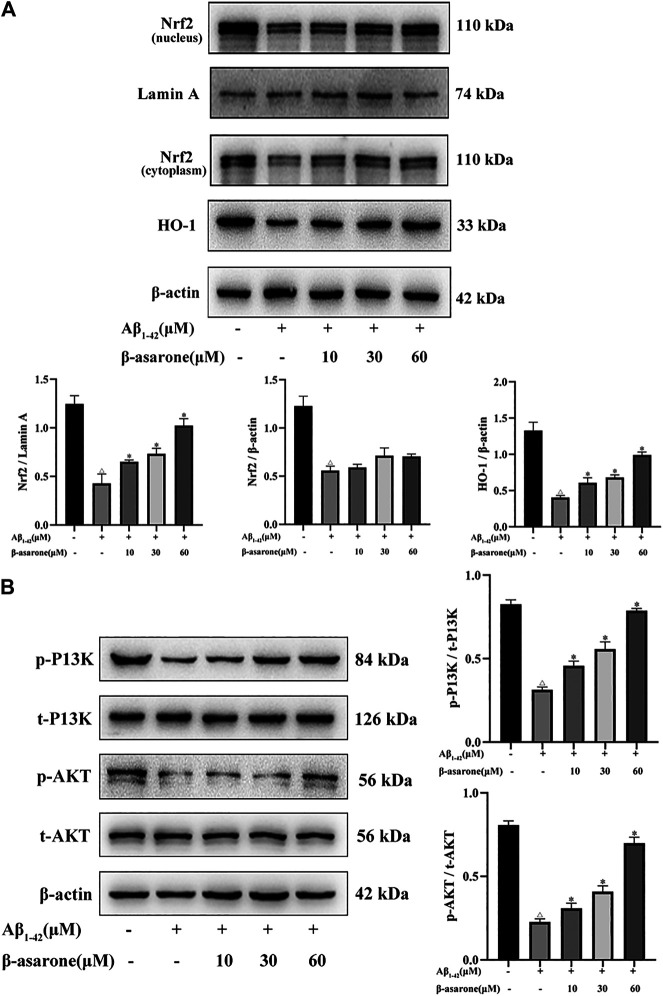
Effect of β-asarone on Aβ_1–42_-induced protein expression of P13K/Akt/Nrf2/HO-1. **(A)** The protein expression levels of Nrf2 in the nucleus and cytoplasm and HO-1 were detected by Western blot. **(B)** The levels of P13K, p-P13K, Akt, and p-Akt were also determined by Western blot. ^△^ *p* < 0.05 compared with the control group; ^*^ *p* < 0.05 compared with the Aβ_1–42_ alone treatment group. The data were expressed as mean ± SD from three independent experiments.

We examined Nrf2 and HO-1 expression levels in PC12 cells by Western blot. As shown in Figure 6A, Aβ suppressed Nrf2 expression in the nucleus and cytoplasm (*p* < 0.05). HO-1 is a key enzyme regulated by Nrf2, and the expression of HO-1 was reduced after Aβ treatment (*p* < 0.05). Compared with the Aβ_1–42_-alone treatment group, the expressions of Nrf2 and HO-1 were increased by β-asarone treatment (*p* < 0.05). Moreover, there was significant difference among different doses of β-asarone (*p* < 0.05). However, the expression of Nrf2 in the cytoplasm was not apparently changed after β-asarone treatment (*p* > 0.05).

Next, we investigated whether β-asarone regulates Aβ_1–42_-stimulated oxidative stress by the P13K/Akt signaling pathway. Compared with the control group, the ratios of phosphorylated to total P13K (p-P13K/t-P13K) and phosphorylated to total Akt (p-Akt/t-Akt) were significantly reduced (Figure 6B; *p* < 0.05). However, the p-P13K/t-P13K and p-Akt/t-Akt ratios were upregulated after β-asarone treatment (*p* < 0.05). Thus, we speculated that β-asarone might regulate Aβ_1–42_-induced oxidative stress by activating the P13K/Akt signaling pathway.

## Discussion

The aggregate of Aβ is the main pathological change in AD (Reitz and Mayeux, 2014). In recent years, several mechanisms of Aβ-mediated neurotoxicity have received intense attention. An increasing number of studies showed that Aβ could contribute to neurodegeneration by generating oxidative stress, mitochondria dysfunction, and cell apoptosis (Crouch et al., 2008; Islam et al., 2019). The rat pheochromocytoma (PC12) cell line has been widely used as an *in vitro* model to evaluate neurotoxicity in AD. Moreover, the cells are particularly sensitive to Aβ-induced injury (Das et al., 2004; Westerink and Ewing, 2008). Therefore, we used an Aβ-stimulated PC12 cell model to explore the neuroprotective effects and potential mechanisms of β-asarone.

Oxidative stress is considered to be an imbalance between the production of ROS and antioxidant systems. Several pieces of evidence suggested that oxidative stress played a crucial role in the development of AD (Chauhan and Chauhan, 2006; Teixeira et al., 2019). The accumulation of Aβ was a contributing factor to ROS overproduction, which would impair antioxidant enzyme activities, causing protein oxidation, lipid peroxidation, and DNA damage (Tong et al., 2018; Islam et al., 2019; Xu et al., 2019). In this study, we found that Aβ_1–42_ treatment increased the levels of ROS and MDA and decreased the levels of SOD, CAT, and GSH-PX. Furthermore, β-asarone could protect PC12 cells against Aβ-induced oxidative damage.

Mitochondria are the main source of ROS. Numerous research studies indicated that Aβ aggregation could cause mitochondria dysfunction such as mitochondrial morphology changes and disruption of mitochondrial membrane potential (Calkins et al., 2011; Radi et al., 2014; Fan et al., 2017). Mitochondria dysfunction could elicit excessive ROS production, resulting in oxidative stress (Tobore, 2019). Increased ROS, in turn, trigger mtDNA damage and the decrease in the mitochondrial membrane potential and cell apoptosis (Radi et al., 2014). In this study, when PC12 cells were exposed to Aβ_1–42_, the mitochondrial membrane potential decreased and mitochondrial ROS increased, but β-asarone reversed these changes.

Apoptosis is a programmed cell death which is mediated by the extrinsic and the intrinsic pathway. The intrinsic pathway is also known as the mitochondria-dependent pathway. When the pathway is triggered, the loss of mitochondrial membrane potential and the release of apoptotic factors occur. Bcl-2 family proteins control the apoptotic mitochondrial events. The imbalance of proapoptotic and antiapoptotic factors promotes the release of cytochrome C, which will eventually lead to caspase activation and induce cell apoptosis (Brenner and Mak, 2009; Radi et al., 2014). Consistent with previous research studies, our results also showed that the imbalance of Bcl-2 and Bax was involved in Aβ-induced apoptosis (Fan et al., 2017; Tong et al., 2018; Xu et al., 2019). Notably, β-asarone may ameliorate mitochondria dysfunction and cell apoptosis via regulation of the Bcl-2 family protein expression.

Nrf2 is considered a crucial sensor that plays a part in the regulation of oxidative stress. A growing number of research studies indicated that overproduced ROS led to the inactivation of Kelch-like ECH-associated protein 1 (Keap1), translocation of Nrf2 into the nucleus, and the combination of antioxidant response elements (AREs). These events ultimately resulted in upregulation of antioxidant enzymes, such as HO-1 (Motohashi et al., 2004; Raghunath et al., 2018; Liang et al., 2019; Xu et al., 2019). HO-1 is an important antioxidant enzyme regulated by Nrf2 (Yu et al., 2019). Nrf2 activation also exerted a neuroprotective effect on the mitochondrial structure and functions in AD (Dong et al., 2020). Previous studies demonstrated that Nrf2 was activated by the P13K/Akt signaling pathway (Kwon et al., 2015; Zhang et al., 2019; Zhuang et al., 2019). In our study, the results showed that β-asarone promoted the expressions of Nrf2 and HO-1 by upregulating the levels of PI3K/Akt phosphorylation. These findings indicated that β-asarone could suppress oxidative stress by modulating the P13K/Akt/Nrf2 signaling pathway.

**FIGURE 7:**
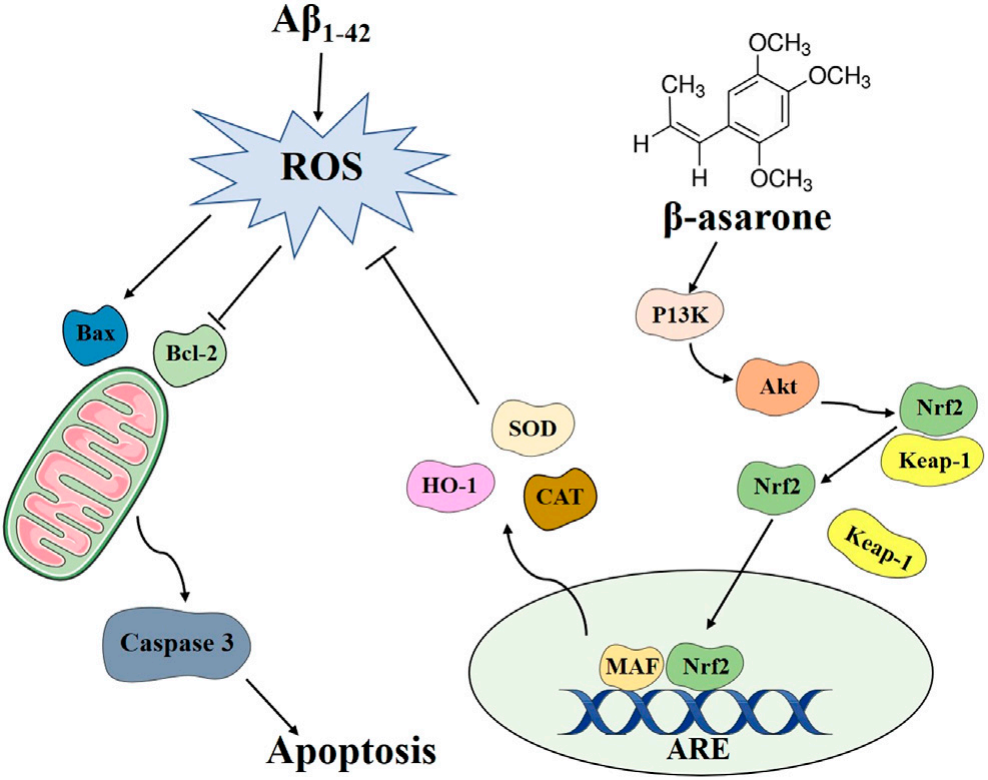
Schematic diagram of the possible mechanism underlying the neuroprotective effects of β-asarone on PC12 cells.

In summary, our study demonstrated that β-asarone could protect Aβ-induced PC12 cells against oxidative stress, mitochondria dysfunction, and cell apoptosis. We describe a possible molecular mechanism by which β-asarone exerts its neuroprotective effects in PC12 cells in Figure 7. Therefore, β-asarone might be a promising therapy for AD.

## Data Availability

The datasets analyzed in this article are not publicly available. Requests to access the datasets should be directed to szypengwei@163.com.
